# Unsupervised item response theory models for assessing sample heterogeneity in patient-reported outcomes measures

**DOI:** 10.1007/s11136-023-03560-5

**Published:** 2023-12-21

**Authors:** Tolulope T. Sajobi, Ridwan A. Sanusi, Nancy E. Mayo, Richard Sawatzky, Lene Kongsgaard Nielsen, Veronique Sebille, Juxin Liu, Eric Bohm, Oluwagbohunmi Awosoga, Colleen M. Norris, Stephen B. Wilton, Matthew T. James, Lisa M. Lix

**Affiliations:** 1 Department of Community Health Sciences, University of Calgary, 3280 Hospital Drive NW, Calgary, T2N 4Z6 Canada; 2 Department of Community Health Sciences, University of Manitoba, Winnipeg, Canada; 3 Center for Outcomes Research and Evaluation (CORE), Research Institute of the McGill University Health Center, McGill University, Montreal, Canada; 4 School of Nursing, Trinity Western University, Langley, British Columbia Canada; 5 Centre for Advancing Health Outcomes, University of British Columbia, Vancouver, Canada; 6 Quality of Life Research Center, Odense University Hospital, Odense, Denmark; 7 Department of Hematolgy, Gødstrup Hospital, Herning, Denmark; 8 Nantes Université, Université de Tours, CHU Nantes, INSERM, methodS in Patient-Centered Outcomes and HEalth ResEarch, SPHERE, 44000 Nantes, France; 9 Department of Mathematics & Statistics, University of Saskatchewan, Saskatoon, Canada; 10 Department of Surgery, University of Manitoba, Winnipeg, Canada; 11 Faculty of Health Sciences, University of Lethbridge, Lethbridge, Canada; 12 Faculty of Nursing, University of Alberta, Edmonton, Canada; 13 Department of Cardiac Sciences, University of Calgary, Calgary, Canada; 14 Department of Medicine, University of Calgary, Calgary, Canada

**Keywords:** Differential item functioning, Hospital anxiety and depression scale, Item response theory, Classification and regression trees, Patient-reported outcomes, Coronary artery disease

## Abstract

**Purpose:**

Unsupervised item-response theory (IRT) models such as polytomous IRT based on recursive partitioning (IRTrees) and mixture IRT (MixIRT) models can be used to assess differential item functioning (DIF) in patient-reported outcome measures (PROMs) when the covariates associated with DIF are unknown a priori. This study examines the consistency of results for IRTrees and MixIRT models.

**Methods:**

Data were from 4478 individuals in the Alberta Provincial Project on Outcome Assessment in Coronary Heart Disease registry who received cardiac angiography in Alberta, Canada, and completed the Hospital Anxiety and Depression Scale (HADS) depression subscale items. The partial credit model (PCM) based on recursive partitioning (PCTree) and mixture PCM (MixPCM) were used to identify covariates associated with differential response patterns to HADS depression subscale items. Model covariates included demographic and clinical characteristics.

**Results:**

The median (interquartile range) age was 64.5(15.7) years, and 3522(78.5%) patients were male. The PCTree identified 4 terminal nodes (subgroups) defined by smoking status, age, and body mass index. A 3-class PCM fits the data well. The MixPCM latent classes were defined by age, disease indication, smoking status, comorbid diabetes, congestive heart failure, and chronic obstructive pulmonary disease.

**Conclusion:**

PCTree and MixPCM were not consistent in detecting covariates associated with differential interpretations of PROM items. Future research will use computer simulations to assess these models’ Type I error and statistical power for identifying covariates associated with DIF.

**Supplementary Information:**

The online version contains supplementary material available at 10.1007/s11136-023-03560-5.

## Introduction

Patient-reported outcomes measures (PROMs) are multi-item questions that elicit patients’ appraisals of their health status and quality of life [1, 2]. PROMs are useful for evaluating treatment efficacy in clinical trials from a patient perspective and comparing population groups for quality improvement [3–5]. Comparing PROM scores among population subgroups relies on the assumption that the measurement model, which describes the relationship between the observed items and the latent construct being measured, is equivalent across these subgroups [6, 7]. This is generally of interest when PROMs are used in potentially heterogeneous populations where respondents may differ in how they interpret and respond to questions about their health and quality of life, a phenomenon known as differential item functioning (DIF). DIF arises when heterogeneity in interpretation and response to the PROM questions are associated with patient characteristics unrelated to the construct of interest being measured [8]. When DIF is ignored in PROM items, the estimated distribution of the PROM scores across population subgroups is biased. Failure to account for DIF in PROM items could affect inferences about PROM scores and their use for supporting decisions in healthcare [8–10]. For example, if patient subgroups consistently provide lower ratings on items of a depression PROM than other subgroups based on their socio-demographic characteristics, this could result in biased estimates of the between-group difference in PROM scores. Incorrect inferences about the meaning of the PROM scores can arise and affect clinical and health policy decisions. This, in turn, could lead to missed opportunities to address pertinent health issues for patients during routine physician visits and reduced access to mental health services.

Existing methods to test for DIF in PROM are mainly group-based methods that assume potentially relevant differences in the target populations are known a priori and can be explained by observed variables such as socio-demographics or health status [11–16]. Also, these multigroup methods evaluate DIF in PROMs items one observed variable at a time. Applying these methods to test for DIF in PROM items in heterogeneous populations where unknown or multiple interacting variables could explain DIF may become onerous with an increasing number of variables.

Unsupervised item response theory (IRT) [16–21] models, which combine IRT models with unsupervised learning methods (e.g., recursive partitioning or mixture models), are an alternative class of IRT models that overcome this limitation by identifying subgroups of patients with different patterns of DIF when patient characteristics associated with DIF are not known a priori*.* These models include IRT models based on the recursive partitioning method (IRTree) and mixture IRT (MixIRT) models. MixIRT model, first proposed by Rost [17], combines latent class models with an IRT modeling framework to identify latent classes across which the IRT parameters are non-invariant. MixIRT models have also been applied to test for DIF [22–25] but can be challenging to implement because of model identification issues [20]. On the other hand, IRTree models such as the Rasch trees [18], polytomous Rasch trees [19], and item-focused trees [20, 21], have been developed to identify DIF items when the variables associated with DIF are not known a priori. With these methods, there is no need to specify variables associated with DIF a priori because they are automatically detected using a data-driven approach.

To date, there has not been any investigation of the comparative performance of IRTree and MixIRT models for detecting DIF in PROMs. The aim of our study was to investigate the consistency of results for these two models. Since these two methods differ in their approach to evaluating MI, we hypothesize that these two methods will be consistent in detecting the presence of heterogeneity but will differ with respect to the number of homogeneous subgroups identified. The manuscript is organized as follows. “Methods” section describes these models and compares their statistical properties. “Numeric example” section applies these models to data from a clinical registry of patients with coronary artery disease who received cardiac angiograms. “Discussion” section discusses the methodological implications of the study findings, the strengths and limitations of the methods, and opportunities for further research.

## Methods

### Partial credit model

Consider a partial credit model (PCM) [26], a polytomous model commonly used for modeling ordinal data, including items comprising PROMs. Let ${Y}_{im}$ denote the $i$ th individual’s response to the $m$ th item. The PCM is defined as,

1 $$P\left( {Y_{im} \ge j| \tau_{mj} ,\theta_{i} } \right) = \frac{{e^{{ - \left( {\tau_{mj} - \theta_{i} } \right)}} }}{{1 + e^{{ - \left( {\tau_{mj} - \theta_{i} } \right)}} }},$$

where $P\left({Y}_{ijm}\ge j| {\tau }_{mj},{\theta }_{i}\right)$ is the $i$ th individual’s probability of response $j$($j$ = 1,…,$J$) on the $m$ th ($m$ = 1,2,…,$M$) item, ${\tau }_{mj}$ denotes the threshold between the (*j*−1)th and *j*th category (*j* = 1,…*, J*) for the $m$ th item, and ${\theta }_{i}$ is the $i$ th patient’s latent factor score, which is often assumed to be distributed as ${\theta }_{i} \sim N$(0,1). While this study considered the PCM, tree-based and mixture models can be generalized to other polytomous IRT models [27].

### Tree-based partial credit model (PCTree)

The PCTree is an unsupervised latent variable model that combines the PCM and recursive partitioning to identify subgroups for which the PCM parameters differ. That is, the PCTree uses input covariates to repeatedly partition the entire sample into homogenous subgroups with respect to the model parameters. Komboz et al. [20] developed a 4-step approach for implementing a PCTree [18]:

1. In Step 1, the PCM is fitted to the entire sample, and the model parameters are estimated via conditional likelihood estimation.
2. In Step 2, the stability of item threshold parameters is assessed for each covariate by conducting structural change tests. Each structural change test involves ordering the contributions of each study respondent to the joint loglikelihood score function of the PCM model for each covariate. DIF is detected, for a covariate, if the ordering of the structural change test statistics for all possible cut-points on that covariate exhibits a systematic change in the individual deviations.
3. In Step 3, among all model covariates, the covariate with the smallest *p*-value for the structural change test is selected for splitting the entire sample into two subgroups (i.e., child nodes). After a covariate has been selected for splitting, the optimal cut-point on this covariate is determined by maximizing the partitioned loglikelihood (i.e., the sum of the loglikelihoods for two separate models: one for the observations to the left and up to the cut-point, and one for the observations to the right of the cut-point), over all potential ($r$–1) cut points, where $r$ is the number of possible values on a covariate. For categorical values, there are $r$–1 cut points.
4. In Step 4, Steps 1–3 are repeated recursively in the child nodes until one of two stopping criteria is reached:
  I. *Bonferroni correction criterion* recursive partitioning of the sample stops if no further significant parameter instability exists for any covariates across all subgroups. Given that multiple structural change tests could result in an inflated familywise Type I error, a Bonferroni correction is applied to α, such that ${\alpha }{\prime}={\alpha }{\prime}/m$, where $m$ = number of tests conducted.
  II. *Minimum terminal node size criterion* this involves pre-specifying a minimum sample size for each terminal node. A recommended simple rule of thumb is to set the minimum node size to be 10 times the average number of parameters per item.

### The mixture PCM

The mixture PCM (MixPCM) [17] aims to uncover heterogeneity by allowing model parameters to vary across two or more latent classes^23^ such that:

2 $$P\left( {Y_{ijm} \ge j| \tau_{mjc} ,C = c,\theta_{i} } \right) = \frac{{e^{{ - \left( {\tau_{mjc} - \theta_{i} } \right)}} }}{{1 + e^{{ - \left( {\tau_{mjc} - \theta_{i} } \right)}} }},$$

where the unconditional probability of response *j* to the $m$ th item (irrespective of class membership is.

3 $$\sum\nolimits_{c = 1}^{C} {\pi_{c} P\left( {Y_{ijm} \ge j| c, \tau_{mjc} , \theta_{i} , C = c} \right)} ,$$

where it is assumed that ${\theta }_{i}\sim N(\mathrm{0,1})$ is the latent trait level for the $i$ th patient *i*, ${\tau }_{mjc}$ denotes the threshold between the (*j*-1)th and *j*th category (*j* = 1,…*, J*) for the $m$ th item in the $c$ th class, and ${\pi }_{c}$ is the mixing proportion that defines the relative sizes of the latent classes, and can be explained by sample characteristics (e.g., demographic, or clinical characteristics) such that $\sum_{c=1}^{C}{\pi }_{c}=1$.

The MixPCM is implemented using a four-step approach:

1. In Step 1, a one-class PCM, which assumes no heterogeneity, is fit to the data. The tenability of the unidimensionality assumption can be assessed using exploratory factor analysis using polychoric correlation with GEOMIN rotation [28–30] or parallel analysis [31]. The unidimensionality assumption is considered satisfied if the ratio of the first and second eigenvalues is greater than 3. If unidimensionality is not a tenable assumption, then MixPCM is not appropriate for testing sample heterogeneity in the data. If the assumption of unidimensionality is satisfied, proceed to step 2.
2. For Step 2, specify MixPCM with increasing numbers of latent classes by allowing the PCM threshold parameters to vary across the latent classes while the latent factor means and standard deviations are constrained to be equal for identifiability purposes.
3. In Step 3, determine the optimal number of latent classes for the MixPCM using the Bayesian Information Criterion (BIC) [32, 33], Vuong-Lo-Mendel-Rubin likelihood ratio test (VLMR)[3, 34], bootstrap likelihood ratio test, and model entropy. The VLMR is used to compare the goodness of fit of models with k, and (k + 1) latent classes; a non-significant VLMR test (*p* > 0.05) prefers the model with the smaller number. Model entropy is used to assess the certainty of class membership (values > 0.8 indicate high confidence in latent class assignment [35]). For the BIC, the optimal model has the smallest BIC value.
4. For the final step, the association of covariates with the estimated latent class membership is explored either via a one-step approach or a three-step approach [35, 36]. In the former, the known covariates are incorporated into the mixture IRT modeling to estimate the posterior probability of latent class membership, conditional on the covariates. The effects of the covariate on class membership are estimated simultaneously, along with the class-specific item parameters. The MixIRT modeling estimates the posterior probability of latent class membership based on the item response data in the three-step approach. In the second step, the class membership is derived based on the most probable posterior probability of class assignment. In the third step, the covariate effects on class membership are estimated using multinomial logistic regression with pseudo draws to account for imperfect classification is used to estimate the covariate effects.

## Numeric example

### Data source

The consistency between the MixPCM and PCTree was examined by analyzing existing population-based data. Data were from the Alberta Provincial Project for Outcome Assessment in Coronary Heart Disease (APPROACH) registry, a population-based database of all adults who received cardiac catheterization in Alberta, Canada [37]. The APPROACH registry maintains one of the most comprehensive data repositories of individuals with coronary artery disease (CAD). The registry includes detailed data on patients’ demographic and clinical characteristics. This registry was chosen because (1) it is made up of heterogeneous CAD patients with varying degrees of CAD severity, different types of treatments received, different experiences with the healthcare system, and diverse demographic and behavioral characteristics, and (2) collects both generic and cardiac-specific patient-reported HRQOL measures. The Hospital Anxiety and Depression Scale (HADS) was selected as a PROM to be investigated for potential DIF effects. Our choice of the HADS for this study was motivated by the unidimensional nature of the HADS subscales (i.e., anxiety and depression subscales) and its excellent psychometric properties for screening for depression in individuals with CAD [38, 39]. The HADS is a self-administered 14-item generic measure of psychological distress comprising two subscales: depression and anxiety [40]. The response options for the HADS items range from zero to three: higher scores indicate more severe depression and/or anxiety. We limited our attention to the depression subscale items.

The study cohort included all adult Alberta residents who (1) underwent a first cardiac catheterization between January 1, 2002, and December 31, 2017, (2) had at least 1-vessel CAD (Duke Coronary Index between 3 and 13), and (3) completed the HADS two weeks after the procedure. In addition to the HADS, data were collected on demographic characteristics (sex, age), multiple comorbid conditions, disease severity, and coronary angiography results. Ethics approval for this study was obtained from the University of Calgary Conjoint Health Research Ethics Board (REB15-1195).

### Statistical analyses

Descriptive statistics were used to summarize the patient’s demographic and clinical characteristics. The assumption of the unidimensionality for the depression items of the HADS was evaluated using parallel analyses and several goodness-of-fit statistics [30, 41–45], including the information-weighted fit mean square error statistic (Infit MNSQ), outlier-sensitive fit statistic (Outfit MNSQ), root mean square error of approximation (RMSEA), comparative fit index (CFI), and standardized root mean square residual (SRMSR). An item with infit MNSQ or outfit MNSQ outside the 0.5–2.0 range is considered a misfit to the PCM [42].

The PCTree and MixPCM were used to identify subgroups of patients with different patterns of DIF or no DIF. Patients’ socio-demographics [sex and age (< 75 years vs$.\ge$ 75 years)] and clinical characteristics (procedure indication, smoking status, body mass index (BMI), and comorbid conditions) were selected as covariates. Several studies have examined the presence of DIF in HADS items for patient’s demographic characteristics, such as age and sex [46–48]. In particular, previous studies have reported age differences in quality of life and risk of adverse health outcomes in elderly ($\ge$ 75 years) heart disease patients compared to younger (< 75 years) patients [49–51]. Although there is a limited investigation of DIF in patient-reported HADS item responses with respect to their clinical and disease characteristics, these patient characteristics are known risk factors for depressive symptoms in CAD patients [51–54].

For the PCTree model, the minimum sample size for each terminal node was set at 250 as a stopping criterion for the recursive partitioning, which also allows for a sufficient sample size for within-node parameter estimation [20]. To facilitate comparability of the models, the covariates were simultaneously incorporated into the MixPCM to estimate class-specific model parameters and the effects of the covariates on latent class membership. Finally, for each method, multinomial logistic regression models were used to test the covariates (i.e., patients’ demographic and disease characteristics) associated with the identified subgroups.

The PCTree analysis and other analyses were implemented in R software [55], while the MixPCM was implemented in Mplus v8.1 [56]. Statistical significance for the analyses was set at $\alpha$= 0.05, except when stated otherwise.

### Results

Table 1 describes the patient characteristics. Of the 4478 patients who completed the HADS, 3522 (78.7%) were male, and 815 (18.2%) were 75 years or older. The majority of patients (69.3%) had acute coronary syndrome as the clinical disease. Hypertension and hyperlipidemia were the most frequent comorbid conditions. About 75% of patients endorsed “often,” on ‘*I can laugh and see the funny side of things*’ and ‘*I can enjoy a good book or radio or TV program*’ items. In contrast, less than 5% of the patients endorsed “very seldom,” on “I can laugh and see the funny side of things”, “I look forward with enjoyment to things”, or “*I can enjoy a good book or radio or TV program*” (Online Table A1). Given that there were a number of sparse response categories, those categories endorsed by less than 1.5% of the sample were merged with the adjacent response categories.

**Table 1:** Characteristics of the study cohort (*N* = 4478)

Characteristic	*N*(%)
Sex (male)	3522(78.7)
Age ($\ge$ 75 years)	815(18.2)
Body mass index (median, IQR)	28.1(6.0)
Procedure indication	
Acute coronary syndrome	3102(69.3)
Stable angina	1376(30.7)
Complex CAD (left main & 3-vessel disease)	1222(27.3)
Current smoker	1062(23.7)
Comorbid conditions	
Diabetes	218(4.9)
Prior myocardial infarction	448(10.0)
Chronic obstructive pulmonary disease	594(13.3)
Hypertension	3136(70.0)
Peripheral vascular disease	311(6.9)
Congestive heart failure	256(5.7)
Hyperlipidemia	3423(76.4)
Cerebrovascular disease	214(4.8)

*IQR* interquartile range, *CAD* coronary artery disease

The conventional one-class PCM provided a good fit for the data. Specifically, the item Infit MNSQ and Outfit MNSQ values were well within the recommended 0.5–2.0 interval (Online Table A2). Additionally, parallel analysis reveal a dominant principal factor; the ratio of the first and second principal factors was approximately 30.2 and acceptable RMSEA, CFI, and SRMSR values, suggesting that the assumption of unidimensionality of the HADS depression items was satisfied (Online Tables A2 & A3).

**Fig. 1:**
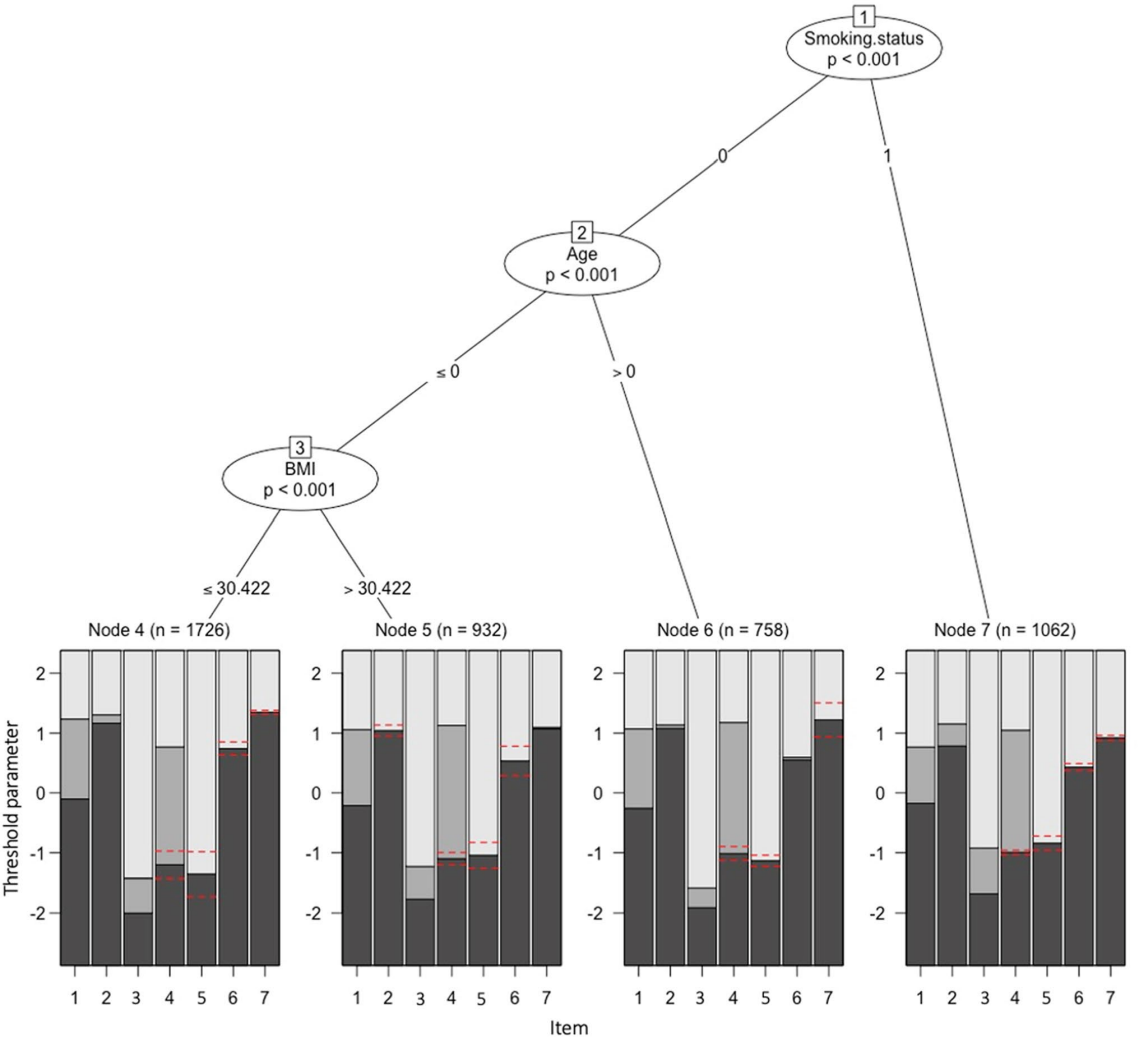
IRTree model for HADS depression subscale items, *N* = 4478. Age = 0 (< 75 years) or 1 ($\ge$ 75 years); smoking status = 0 (Non-smoker) or 1 (crrent smoker); *BMI* body mass index; Red dashed lines = items with reversed thresholds; region plots (dark and light grey regions) = regions of most probable category responses of an item. They are depicted for each item with the estimated threshold parameters of the partial credit model in the corresponding node. (Color figure online)

The PCTree identified four terminal nodes (i.e., subgroups) of patients defined by the interaction among smoking status, age, and BMI (Fig. 1). The entire sample was first partitioned using the smoking status variable, indicating that this was the most important variable that explained sample heterogeneity in the HADS depression subscale items. The first terminal node, which accounted for 23.7% of the sample, consisted of current smokers. The second terminal node (16.9%) included non-smokers older than 75. The third terminal node (20.8%) was comprised of older (i.e., > 75 years) non-smokers with BMI > 30.4, while the final terminal node (38.5%) consisted of patients at most 75 years and non-smoking with BMI $\le$ 30.4. The region plots in these terminal nodes of the PCTree model in Fig. 1 show patterns of differences in the HADS items and item response categories for which patients had inconsistent patterns of responses. For example, for item #2 (“*I can laugh and see the funny side of things*”), the region of the second category, shaded in the second darkest gray color, was largest for patients who are smokers and lowest for non-smoking patients who are < 75 years and with a BMI > 30.422. Similarly, for item 2 (*I feel cheerfu*l), the region of the second category, shaded in the second darkest gray color, was largest for smokers and lowest for non-smoking patients < 75 years. Results from multinomial logistic regression analysis revealed that the variance inflation factors were all < 5, which indicates the absence of multicollinearity among the covariates. Significant differences exist among the terminal nodes with respect to sex, procedure indication, disease complexity, diabetes, hyperlipidemia, myocardial infarction, cerebrovascular disease, chronic obstructive pulmonary disease (COPD), and hypertension (Table 2).

**Table 2:** Adjusted odds ratio [95% confidence interval] for PCTree model subgroups and patient characteristics

Patients’ characteristic	Subgroup 2 vs. 1	Sublgroup 3 vs. 1	Subgroup 4 vs. 1
Sex (Female)	0.77[0.61, 0.96]^*^	1.07[0.86, 1.33]	1.27[1.04, 1.53]^*^
Age (> 75 years)	–	–	–
Body mass index (median, IQR)	–	–	–
Procedure indication (stable Angina)	0.46[0.37, 0.58]^*^	0.38[0.31, 0.47]^*^	0.38[0.31, 0.46]^*^
Current smoker	–	–	–
Complex CAD (Left main & 3-vessel disease)	1.89[1.53, 2.34]^*^	1.06[0.86, 1.31]	1.18[0.98, 1.42]
Comorbid conditions			
Diabetes	0.84[0.51, 1.39]	2.34[1.56, 3.51]^*^	1.17[0.77, 1.77]
Prior myocardial infarction	2.10[1.55, 2.86]^*^	0.97[0.69, 1.35]	1.22[0.92, 1.63]
Chronic obstructive pulmonary disease	0.70[0.53, 0.91]^*^	0.73[0.57, 0.94]^*^	0.44[0.35, 0.56]^*^
Hypertension	1.90[1.52, 2.37]^*^	1.87[1.52, 2.29]^*^	1.05[0.89, 1.24]
Peripheral vascular disease	0.67[0.47, 0.96]^*^	0.75[0.53, 1.04]	0.59[0.44, 0.80]^*^
Congestive heart failure	2.20[1.52, 3.19]^*^	1.11[0.74, 1.66]	0.91[0.63, 1.33]
Hyperlipidemia	0.77[0.62, 0.96]^*^	1.37[1.09, 1.72]^*^	0.93[0.78, 1.12]
Cerebrovascular disease	1.90[1.23, 2.93]^*^	1.07[0.68, 1.70]	1.35[0.90, 2.03]

*CAD* coronary artery disease, *PCTree* tree-based partial credit model; age, body mass index, and current smoker were excluded as predictors since they were used to define the PCTree nodes

*$$p$$< 0.05

For the MixPCM, we fitted one-, two-, and three-class models to the data; models with more classes could not be fitted to the data due to model identification problems. A three-class model provided an optimal fit to the data based on the BIC and a VLMR test comparing two-class and three-class models (Table 3). The classes consisted of 1609 (36.0%), 2145(48.0%), and 715(16.0%) patients, respectively. The multinomial logistic regression models revealed significant differences among the classes on age, sex, smoking status, procedure indication, and comorbid conditions. Patients in class 2 had lower odds of presenting with stable angina, being current smokers, and having comorbid diabetes, prior myocardial infarction, COPD, congestive heart failure, and cerebrovascular disease than patients in class 1. Patients in class 3 had higher odds of being older (> 75 years) but lower odds of being current smokers, having COPD, and having cerebrovascular disease than patients in class 1 (Table 4).

**Table 3:** Fit statistics for MixPCM with 1 to 3 latent classes for the HADS depression subscale items (*N* = 4478)

Fit statistics	1-Class	2-Class	3-Class
2 × Loglikelihood	− 26,582.9	− 23,967.4	− 22,900.0
Bayesian information criterion	53,259.9	47,525.3	46,481.0
Entropy	–	0.86	0.79
Vuong–Lo–Mendel–Rubin likelihood ratio test (*p*-value)	–	< 0.01	< 0.01
Bootstrap likelihood ratio test	–	< 0.01	< 0.01
Class proportion			
Class 1	1.00	0.64	0.36
Class 2	–	0.36	0.48
Class 3	–	–	0.16

*MixPCM* mixture partial credit model, *HADS* hospital anxiety and depression scale

**Table 4:** Adjusted odds ratio [95% confidence interval] for three-class MixPCM and patient characteristics

Characteristic	Class 2 vs. 1	Class 3 vs. 1
Sex (female)	1.19[0.98, 1.49]	1.14[0.94, 1.45]
Age (> 75 years)	1.02[0.80, 1.31]	1.31[1.02, 1.69]^*^
Body mass index (median, IQR)	0.99[0.98, 1.00]	1.00[0.99, 1.01]
Procedure indication (stable angina)	1.21[1.15, 1.70]^*^	1.03[0.88, 1.32]
Current smoker	0.44[0.36, 0.53]^*^	0.60[0.49, 0.74]^*^
Complex CAD (left main)	0.89[0.73, 1.08]	1.01[0.82, 1.23]
Comorbid conditions		
Diabetes	0.61[0.41, 0.90]^*^	0.90[0.61, 1.32]
Prior myocardial Infarction	0.59[0.44, 0.78]^*^	0.78[0.59, 1.03]
Chronic obstructive pulmonary disease	0.70[0.55, 0.90]^*^	0.78[0.61, 0.99]^*^
Hypertension	1.06[0.87, 1.29]	1.12[0.96,1.44]
Peripheral vascular disease	0.90[0.64, 1.26]	1.13[0.80, 1.59]
Congestive heart failure	0.65[0.46, 0.93]^*^	0.78[0.55, 1.12]
Hyperlipidemia	1.06[0.87, 1.31]	1.12[0.90, 1.39]
Cerebrovascular disease	0.50[0.35, 0.71]^*^	0.54[0.37, 0.78]^*^

*CAD* coronary artery disease, *IQR* interquartile range, *MixPCM* mixture partial credit model

*$$p$$< 0.05

## Discussion

This study investigates the extent to which PCTree and MixPCM consistently identify patient covariates associated with different interpretations of HADS Depression items. Our analyses show that both models identified age and smoking status (i.e., whether a patient was a current smoker) as covariates associated with DIF. Overall, the PCTree model identified four subgroups of patients defined by smoking status, age, and BMI. However, MixPCM identified three latent classes defined by age, smoking status, procedure indication, and multiple comorbid conditions.

There are several similarities and notable differences in the properties of these two models and how they are operationalized to evaluate sample heterogeneity (Table 5). Both are similar concerning the underlying assumption of unidimensionality of the data, large sample size requirements, and unsupervised learning approaches for DIF detection. Unlike existing group-based methods designed to detect PROM items that exhibit DIF, these unsupervised latent variable models present a global approach for identifying individuals that exhibit DIF instead of the items that exhibit DIF. These methods are particularly of interest in routine clinical practice where PROMs data help inform clinical decisions (e.g., treatment strategies, goals of care, referral for additional services, and so on) about a patient’s care. Identifying individuals with a propensity for DIF can help clinicians contextualize each patient’s responses to PROMs, support shared decision-making, and inform the delivery of personalized disease management. However, these methods have notable differences. First, these models differ with respect to the evaluation of sample heterogeneity. The PCTree evaluates sample heterogeneity via recursive partitioning of the sample into independent homogeneous subgroups for which the PCM parameters are non-invariant using a set of covariates. MixPCM, on the other hand, evaluates sample heterogeneity by estimating the posterior probability of latent class membership for each individual so that the latent classes are non-invariant for the PCM parameters. Second, selecting the optimal number of latent classes in MixPCM is based on known goodness-of-fit statistics, whereas determining the final subgroups in PCTree depends on the likelihood ratio test used in determining optimal split across known covariates. LRT is known to be sensitive to study sample size [57]. Third, unlike the tree-based IRT model, which requires specifying a set of covariates as input variables, the MixPCM models can estimate the latent subgroups with and without specifying a set of covariates. Finally, there are notable differences in the computational requirements for implementing tree-based IRT models and mixture IRT models. Estimating latent classes from mixture IRT models can be computationally intensive as it involves sequentially fitting multiple models and assessing model fit until an optimal number of latent classes is identified. In addition, MixIRT model parameters are estimated based on numeric computation, which is prone to model convergence issues depending on the number of starting values specified. Implementing tree-based models requires only a few lines of code that are less computationally intensive.

**Table 5:** Comparison of mixture item response theory and tree-based item response theory models

Attributes	Mixture item response theory (MixIRT) model	Polytomous item response theory tree (IRTree) model
Description	This model combines latent class analysis with IRT to identify homogenous subgroups (i.e., latent classes) from the data. The IRT model parameters are allowed to vary across latent classes. Sample heterogeneity is operationalized as differences in IRT parameters across (i.e., latent classes) for which the IRT parameters are non-invariant [17]	A tree-based polytomous IRT model in which the sample is recursively partitioned into homogeneous subgroups. The study sample is partitioned into homogenous subgroups by identifying the most important covariate for which the optimal cut point maximizes the differences in the measurement model (PC Model) parameters across the subgroups [20]. The process is repeated recursively in the child nodes until a stopping criterion is reached
The unidimensionality of the patient-reported outcomes measure (PROM)	Assumes a unidimensional factor structure	Assumes a unidimensional factor structure
Characterization of sample heterogeneity	Can detect sample heterogeneity by incorporating known covariates to Iderive the posterior probability of latent class membership [17]	The IRTree handles heterogeneity with respect to differences in partial credit model parameters in the sample by splitting the sample using known covariates as input variables [20]
	Can also detect unobserved sample heterogeneity without known covariates	Unobserved sample heterogeneity cannot be determined without known covariates
Model overfitting	Fit statistics such as the bootstrap likelihood ratio test internally validate the optimal number of latent classes via bootstrapped LRT [29]	Overfitting is avoided by using adopting a Bonferroni correction when determining the splitting points
Incorporation of multiple covariates	MixIRT can detect sample heterogeneity with and without multiple known covariates [32, 33]	The IRTree can only detect sample heterogeneity using multiple covariates only [20]. IRTree models are sensitive to the type of variable and the number of variables included as input variables
Sample size requirements	Requires large sample sizes to ensure stable parameter estimation [9, 10]	Requires large sample sizes to ensure stable parameter estimation. A simple recommended rule of thumb is 10 times the average number of model parameters per item for each node
Computation efficiency	MixIRT model requires the fitting of multiple models by sequentially increasing the number of latent classes. Multiple fit statistics are often used to determine the optimal number of latent classes. Implementing MixIRT models can be computationally intensive. The model can exhibit model convergence issues even with a large number of starting values	The IRTree does not require fitting multiple models before determining the optimal number of subgroups. It requires only a few lines of syntax and is not computationally intensive
Model misspecification	Multiple fit statistics, such as Bayesian information criterion (BIC), sample size-adjusted BIC, Bootstrap likelihood ratio test, and Vuong–Lo–Mendell–Rubin likelihood ratio test, are available for determining the optimal number of latent classes in MixIRT [27–31]	Fit statistics for determining the optimal number of subgroups are not available
Software implementation	Implemented in MPlus [50] and R software *psychomix* package [50]	Implemented using the R software package *psychotree *[50]

*MixIRT* mixture item response theory model, *PCTree* tree-based partial credit model, *DIF* differential item functioning

Tree-based latent variable models, such as PCTree, are promising methods for identifying sample heterogeneity in PROMs in heterogeneous population of patients defined by multiple interacting variables. Unlike conventional group-based methods for DIF detection that require a priori specification of the variable associated with DIF, these methods can be appealing for handling population heterogeneity in PROM scores. They can be used in exploratory analyses to generate hypotheses about potential DIF variables.

Despite the strengths of these models, they are prone to the inherent limitations of unsupervised learning methods and latent variable methods from which they are derived. Specifically, tree-based models are prone to overfitting, which may lead to the detection of spurious subgroups. Bonferroni-corrected structural change tests and pre-specification of minimum terminal node size are two recommended approaches for preventing model overfitting in tree-based models. Furthermore, the accuracy of the tree-based IRT models for detecting sample heterogeneity depends, to a greater extent, on the variables included as input covariates. For example, the conclusions from the empirical analysis in this study are limited to the available demographic, clinical, and disease characteristics used as input variables. The APPROACH registry does not collect data on a history of depression, medical treatment for depression, cognitive impairment, and other important risk factors that may be associated with DIF in patient-reported HADs items. This limits the generalizability of the conclusions from this empirical study. Moreover, changing the type (i.e., ordinal, continuous, or mixed) and the number of covariates included in the model could influence the number and type of homogenous subgroups (nodes) identified.

Future research could investigate determining the optimal minimum sample size requirement for the terminal nodes across various data characteristics. Also, comparing PCTree and latent class PCM models was based on a single empirical data. Although results from simulation studies reported by Komboz et al. [20] show that PCTree exhibit comparable control of familywise Type I error as the multigroup PCM, the comparison of the Type I error of PCM and MixPCM is yet to be investigated. Future research will use computer simulations to examine the comparative performance of PCTree and MixPCM for detecting DIF in PROM items, with respect to their Type I error and statistical power, under a variety of distributional and data characteristics. Finally, the empirical comparison of these unsupervised learning methods in this study focuses on identifying homogeneous subgroups of individuals consistent patterns of responses to the HADS items and not detecting HADS items that exhibit DIF. While mixture IRT models have been extended to detect DIF and estimate DIF effect sizes in PROM items [58, 59], future research will investigate the extension of tree-based IRT models for detecting DIF PROM items.

## Conclusion

In summary, this study revealed that MixPCM and PCTree models are inconsistent in identifying covariates associated with DIF in PROM items. While PCTree is an alternative methodology to the mixture IRT model for examining sample heterogeneity in PROMs items, future research is needed, including computer simulations to evaluate the Type I error and statistical power of these models for DIF detection.

### Supplementary Information

Below is the link to the electronic supplementary material.

Supplementary file1 (DOCX 22 KB)

Supplementary file2 (DOCX 20 KB)

## Data Availability

The data used for these analyses are not openly available due to reasons of sensitivity and are only available from the corresponding author upon reasonable request and with permission from the University of Calgary Conjoint Health Research Ethics Board and the APPROACH Research Group at the University of Calgary.
