# Supplementary material for: Unsupervised item response theory models for assessing sample heterogeneity in patient-reported outcomes measures
Source: Qual Life Res. 2023 Dec 21;33(3):853–64. doi: 10.1007/s11136-023-03560-5 (PMC10894181; doi:10.1007/s11136-023-03560-5)
Supplement: Supplementary file 2 — Supplementary file2 (DOCX 20 KB) [file 11136_2023_3560_MOESM2_ESM.docx]

## **APPENDIX**

**Table A1: Frequency (%) of patients’ responses to the HADS depression subscale items (N = 4,478)**

| **Item** | **Response Categories** | | | |
| --- | --- | --- | --- | --- |
|  | Often | Sometimes | Not Often | Very Seldom |
| 1. I still enjoy the things I used to enjoy | 2053(45.8) | 1939(43.3) | 351(7.8) | 135(3.0) |
| 2. I can laugh and see the funny side of things | 3374(75.3) | 904(20.2) | 185(4.1) | 15(0.3) |
|  | Very Seldom | Not Often | Sometimes | Often |
| 3. I feel cheerful | 25(0.6) | 167(3.7) | 1096(24.5) | 3190(71.2) |
|  | Often | Sometimes | Not Often | Very Seldom |
| 4. I feel as if I am slowed down | 363(8.1) | 896(20.0) | 2530(56.5) | 689(15.4) |
| 5. I have lost interest in my appearance | 88(2.0) | 365(8.2) | 986(22.0) | 3039(67.9) |
| 6. I look forward with enjoyment to things | 2880(64.3) | 1112(24.8) | 430(9.6) | 56(1.3) |
| 7. I can enjoy a good book or radio, or TV program | 3506(78.3) | 783(17.5) | 133(3.0) | 56(1.3) |

Note: HADS = Hospital anxiety and depression scale

**Table A2: Item Threshold Parameters and Fit Statistics for one-class PCM**

| **HADS Depression Subscale Items** | **Threshold 1** | **Threshold 2** | **Threshold 3** | **Infit MNSQ** | **Outfit MNSQ** |
| --- | --- | --- | --- | --- | --- |
| **Initial Model Parameter Estimates and Fit Statistics** | | | | | |
| 2. I still enjoy the things I used to enjoy | -0.33 | 1.64 | **3.46** | 0.81 | 0.83 |
| 4. I can laugh and see the funny side of things | 0.97 | 3.22 | 6.13 | 0.69 | 0.55 |
| 6. I feel cheerful | 0.77 | 3.11 | 5.62 | 0.73 | 0.68 |
| 8. I feel as if I am slowed down | -2.21 | -1.22 | -0.04 | 1.56 | 1.57 |
| 10. I have lost interest in my appearance | -3.04 | -2.32 | -1.60 | 1.18 | 1.15 |
| 12. I look forward with enjoyment to things | 0.39 | 2.22 | 4.55 | 0.70 | 0.67 |
| 14. I can enjoy a good book or radio, or TV program | 1.18 | 3.28 | 5.17 | 0.79 | 0.67 |
| **Revised Model Parameter Estimates and Fit Statistics** | | | | | |
| 2. I still enjoy the things I used to enjoy | -0.33 | 1.65 | 3.48 | 0.82 | 0.84 |
| 4. I can laugh and see the funny side of things | 0.96 | 3.31 | - | 0.69 | 0.56 |
| 6. I feel cheerful | 0.75 | 3.23 | - | 0.73 | 0.68 |
| 8. I feel as if I am slowed down | -2.22 | -1.23 | -0.03 | 1.57 | 1.57 |
| 10. I have lost interest in my appearance | -3.04 | -2.32 | -1.60 | 1.19 | 1.15 |
| 12. I look forward with enjoyment to things | 0.37 | 2.36 | - | 0.71 | 0.67 |
| 14. I can enjoy a good book or radio, or TV program | 1.13 | 3.44 | - | 0.79 | 0.67 |

Note: PCM = partial credit model; ‘-‘ corresponds to response categories merged with the adjacent responses; Revised model was based on data where the response categories with low frequencies were collapsed with the adjacent ones; Infit MNSQ: Information-weighted fit statistic mean square error; Outfit MNSQ: Outlier-sensitive fit statistic mean square error.
